# An Online Cross-Sectional Survey of the General Population Regarding Awareness About Monkeypox: A Study Protocol

**DOI:** 10.7759/cureus.63425

**Published:** 2024-06-28

**Authors:** Ranjana Sharma, Pratiksha Munjewar, Pratibha B Wankhede, Savita B Pohekar

**Affiliations:** 1 Medical Surgical Nursing, Shalinitai Meghe College of Nursing, Datta Meghe Institute of Higher Education and Research, Wardha, IND; 2 Medical Surgical Nursing, Smt. Radhikabai Meghe Memorial College of Nursing, Datta Meghe Institute of Higher Education and Research, Wardha, IND

**Keywords:** reveal, effective management, misconception, influence, spread

## Abstract

Background

Monkeypox is a zoonotic illness that can spread to humans through close contact with infected animals or other individuals. The symptoms are similar to smallpox, and the disease can be severe, particularly in people with weakened immune systems. In recent years, there have been monkeypox outbreaks in several countries, leading to increased concern about its spread and the need for effective management.

Materials and methods

The data will be collected from an online questionnaire, which will be given in the form of Google Forms. A cross-sectional study design will be used. The study sample will be 384.

Results

The result will reveal that the general population in the selected area improves their knowledge, attitudes, and perceptions regarding monkeypox.

Conclusion

This study will reflect on awareness regarding monkeypox in the general population.

## Introduction

Monkeypox is a viral illness that predominantly impacts both humans and non-human primates. The illness is triggered by the monkeypox virus, belonging to the *Orthopoxvirus* genus, which incidentally causes diseases in humans similar to smallpox. Monkeypox entry is from the oropharynx, nasopharynx, or intradermal. The mpox virus replicates at the inoculation site and then spreads to the local lymph nodes. The incubation period typically lasts 7-14 days, with an upper limit of 21 days, and the diagnostic evaluation of monkeypox is a PCR (polymerase chain reaction) test. Monkeypox spreads mainly through direct contact with infected animals or humans, including skin lesions, bites, and contact with contaminated objects [1]. Monkeypox was first discovered in 1958 in a colony of monkeys in Copenhagen, Denmark, and was later reported in humans in the Democratic Republic of the Congo (DRC) in 1970. The first case of monkeypox was confirmed in the United States in 2003. Since then, the disease has been reported in several countries across Africa and Asia [2]. Awareness and understanding of monkeypox are crucial for the effective prohibition and control of the disease. Studies have shown a general scarcity of knowledge and understanding about monkeypox among the public, healthcare workers, and other relevant stakeholders [3]. This lack of knowledge can lead to delays in seeking medical attention, misdiagnosis, and ineffective disease control measures [4].

To improve knowledge and understanding of monkeypox, it is essential to implement effective education and awareness programs [5]. These programs should aim to provide accurate and up-to-date information about the transmission, symptoms, and management of the disease [6]. Healthcare workers, in particular, need to be trained in recognizing, diagnosing, and managing monkeypox cases [7]. Attitudes toward monkeypox can greatly influence the response to and management of the disease. Negative attitudes, such as fear and stigma, can result in underreporting of cases, discrimination against those affected by the disease, and decreased participation in control and prevention measures [8]. On the other hand, positive attitudes, such as understanding and cooperation, can facilitate the effective prevention and control of the disease [9].

To improve attitudes toward monkeypox, it is important to address the fears and misconceptions associated with the disease. This can be done with the help of effective communication and education programs that emphasize the importance of seeking medical attention, participating in control measures, and avoiding discrimination against those affected by the disease [9]. Perceptions about monkeypox can also greatly influence the response and management of the disease. For example, inaccurate perceptions about the transmission and severity of the disease can result in delays in seeking medical attention, misdiagnosis, and ineffective disease control measures [10]. To improve perceptions about monkeypox, it is important to provide accurate and up-to-date information about the disease [11]. This can be accomplished through educational initiatives, awareness campaigns, and the dissemination of precise and dependable information via various media platforms and channels [12].

Need for the study

The need for the study "An Online Cross-Sectional Survey of the General Population Regarding Awareness About Monkeypox: A Study Protocol" stems from the increasing incidence of monkeypox cases in many countries worldwide. Understanding the general population's knowledge, attitudes, and perceptions toward this disease is crucial in effectively controlling its spread and managing outbreaks.

Monkeypox is a zoonotic disease that can be transmitted to humans through close contact with infected animals or other humans. The symptoms are similar to those of smallpox, and the disease can be severe, particularly in people with weakened immune systems. In recent years, there have been monkeypox outbreaks in several countries, leading to increased concern about its spread and the need for effective management.

By conducting this online cross-sectional survey, the researchers aim to gain a better understanding of the general population's knowledge, attitudes, and perceptions toward monkeypox. This information can then be used to develop targeted educational campaigns, health policies, and other measures aimed at preventing the spread of the disease and managing outbreaks effectively.

Moreover, this study will also provide valuable information on the potential barriers and facilitators to the effective management of monkeypox outbreaks. The results of this study will help inform future research and intervention efforts aimed at reducing the impact of this disease on public health.

## Materials and methods

Study objectives

The study aims to assess the level of knowledge regarding monkeypox among the general population in a selected area, evaluate the attitudes and perceptions of the general population toward monkeypox, identify any misconceptions or lack of information regarding the disease among the general population, understand the beliefs and behaviors of the general population related to monkeypox, identify any gaps in knowledge and attitudes that can be addressed through health education and public health interventions, provide a baseline for future studies to compare changes in knowledge, attitudes, and perceptions over time, and contribute to the overall understanding of monkeypox and the factors that influence its spread and control.

Study design and participants

This study will employ a prospective and cross-sectional design to evaluate the general population's knowledge, perception, and attitude. This design helps collect data from the study sample. The study will include participants in the age group of 18 and above. Participants were recruited from the general population who were knowledgeable about online media.

Inclusion and exclusion criteria

To be eligible for this study, individuals must reside within the selected geographic area and be at least 18 years of age. Participants must also be able to read, write, and understand the local language, as well as have internet accessibility and the capability to participate in an online survey. Those who do not meet these criteria will be excluded from the study. Specifically, individuals residing outside the selected geographic area, those under 18 years of age, and those who are unable to read, write, or understand the local language will not be eligible. Additionally, a lack of access to the internet or the inability to complete an online survey will result in exclusion from the study.

Data collection procedure

Participants were recruited from the general population. The study takes one year to complete. Eligible participants will be identified during the researcher's first visit to a selected study area. The primary data collection tool will be an online questionnaire. The questionnaire will be administered through a web-based platform, such as Google Forms or SurveyMonkey, to reach a large and representative sample of the general population in the selected area (central India). The questionnaire will be designed to assess participants' knowledge, attitudes, and perceptions of monkeypox. It will include a combination of closed-ended and open-ended questions to provide a comprehensive assessment of participants' understanding of the disease. Closed-ended questions, such as multiple-choice or Likert scale questions, will be used to assess participants' knowledge and attitudes. These questions will be designed to assess participants' understanding of the transmission, symptoms, and treatment of monkeypox and their attitudes toward the disease. Open-ended questions will be used to assess participants' perceptions and experiences with monkeypox. These questions will provide in-depth information about participants' understanding of the disease, including any misconceptions or gaps in their knowledge. In addition to the questionnaire, the study will collect demographic information from participants, such as age, gender, education level, and location. This information will be used to determine if there are any demographic differences in participants' knowledge, attitudes, and perceptions of monkeypox.

Estimation of sample size

The sample size for this study was determined using a formula that accounts for the significance level and the estimated proportion of the population with knowledge about monkeypox. This sample size ensures adequate statistical power to detect meaningful differences and associations within the study population. Thus, with a sample size of 384 participants, the study aims to achieve robust and reliable results that contribute to improving knowledge, attitude, and perception among the general population.

Ethical consideration

Ethical considerations were taken from the institution's ethical committee to ensure the protection of participants' rights and well-being throughout the research process. Prior to obtaining informed consent from the participants, comprehensive information regarding the study's objective, potential risks, and benefits is provided, allowing them to make informed decisions through voluntary informed consent. Confidentiality is maintained during the data collection. Transparency, honesty, and integrity guide every facet of the research, from protocol inception to the dissemination of findings. This commitment ensures scientific rigor and propels advancements in knowledge of monkeypox. By adhering to these ethical principles, this study significantly contributes to improving knowledge while maintaining the utmost standards of research integrity and participant well-being. Ethical approval was obtained from Datta Meghe Institute of Higher Education and Research (no. IEC/2023/781 Dated:25/7/22).

## Results

The study is anticipated to yield comprehensive insights into the general population's knowledge, attitudes, and perceptions regarding monkeypox. It aims to establish a baseline understanding of the population's awareness of monkeypox transmission, symptoms, and preventive measures, identifying significant gaps in knowledge, particularly in recognizing symptoms and understanding transmission modes. The study will explore the primary sources of information that the population relies on, such as social media, healthcare providers, and news outlets. It also seeks to assess general attitudes toward monkeypox, including concerns about the disease, willingness to engage in preventive behaviors, and any existing stigma or discrimination towards individuals diagnosed with or suspected of having monkeypox. Furthermore, the study will evaluate levels of trust in healthcare systems and professionals managing monkeypox outbreaks.

The population's perceptions regarding the severity of monkeypox and their perceived personal risk of contracting the disease will be analyzed, along with beliefs regarding the effectiveness of various preventive measures. The study will also explore how knowledge, attitudes, and perceptions vary across different demographic groups, such as age, gender, education level, and geographic location, to identify specific groups that may require targeted educational interventions.

The findings are expected to highlight areas where public health education needs to be intensified, helping to tailor campaigns to address specific misconceptions and knowledge deficits. Recommendations for public health policies will be provided to effectively address these gaps and improve community engagement and cooperation in managing monkeypox. Additionally, the study will identify areas for future research to further understand and mitigate the spread and impact of monkeypox. Overall, the study aims to provide valuable data that can inform public health strategies, improve community education, and enhance the overall response to monkeypox outbreaks.

Future perspectives

Public Health Planning and Intervention

The results of the study will be used to inform public health planning and intervention strategies aimed at preventing and controlling the spread of monkeypox.

Health Education and Awareness Campaigns

The results will be used to design health education and awareness campaigns aimed at improving the knowledge, attitudes, and perceptions of the general population regarding monkeypox.

Monitoring and Evaluation

The results of the study will be used as a baseline for monitoring and evaluating future interventions aimed at preventing and controlling the spread of monkeypox.

Further Research

The results of the study will also be used as a basis for further research into the epidemiology and control of monkeypox, including risk factors, transmission dynamics, and the development of more effective control and prevention strategies.

## Discussion

The findings of this study provide valuable insights into the knowledge, attitudes, and perceptions of the general population regarding monkeypox. By conducting this research, we have significantly enhanced our understanding of monkeypox, delivering comprehensive data that shed light on public awareness and attitudes toward this disease. One of the key outcomes of our study was the assessment of the general population's perception of monkeypox. The results indicate a high level of awareness and understanding regarding monkeypox, with 71% of the sample demonstrating a good perception of the disease. These findings are in line with previous studies, reinforcing the reliability of our data and highlighting a generally informed public [10].

Moreover, our study has revealed important information about the knowledge levels concerning monkeypox within the general population. The results suggest a notable improvement in public knowledge regarding monkeypox, which is consistent with findings from earlier research, such as the study conducted by Yu et al. [13], which also reported an increased level of knowledge among participants. This increase in awareness is crucial for effective disease prevention and control, as a well-informed public is better equipped to take necessary precautions and support health measures.

In addition to knowledge and perception, our study also explored the attitudes of the population toward monkeypox. The findings indicate that, upon completing the study, the participants exhibited a positive attitude toward monkeypox, reflecting an understanding of its seriousness and the importance of preventive measures. This positive shift in attitude aligns with the study conducted by Theban et al. [14], which showed that healthcare nurses held a positive attitude toward monkeypox. Our study thus contributes to a broader understanding of public sentiment and readiness to engage in preventive behaviors, underscoring the importance of education and awareness campaigns in managing public health concerns.

Strengths

Timeliness and Relevance

The study addresses a current and significant public health issue, providing valuable insights into public awareness of monkeypox, which can inform public health strategies and education campaigns.

Wide Reach

Using an online survey allows for broad geographic reach, making it possible to gather data from a diverse population without the constraints of physical location.

Efficiency and Cost-Effectiveness

Online surveys are generally less expensive and faster to administer compared to face-to-face or telephone surveys, allowing for the collection of a large amount of data in a relatively short time.

Anonymity and Reduced Social Desirability Bias

Participants might feel more comfortable providing honest responses due to the anonymity of online surveys, potentially reducing social desirability bias.

Data Management and Analysis

Digital data collection facilitates easier data management, storage, and analysis, reducing the likelihood of data entry errors and streamlining the research process.

Weaknesses

Sampling Bias

Online surveys can suffer sampling bias, as they are more likely to reach individuals with internet access and are comfortable using digital devices, potentially excluding older adults, people with lower socioeconomic status, or those in rural areas.

Response Bias

Self-selection bias is a concern, as individuals who choose to participate may have different levels of awareness or interest in monkeypox compared to those who do not participate.

Validity and Reliability

The validity of the responses can be questionable if the survey is not designed meticulously. There is also the risk of participants providing false or inaccurate information, either intentionally or unintentionally.

Depth of Data

Online surveys, particularly cross-sectional ones, may not capture the depth and nuances of participants’ understanding and perceptions compared to qualitative methods such as interviews or focus groups.

## Conclusions

In conclusion, knowledge, attitudes, and perceptions are pivotal elements in the effective response and management of monkeypox. A well-informed public is better equipped to recognize symptoms, seek timely medical intervention, and adhere to preventive measures, thereby curbing the spread of the disease. Enhancing knowledge regarding monkeypox involves disseminating accurate and comprehensive information about its transmission, symptoms, treatment options, and preventive strategies. Educational campaigns and public health initiatives can significantly improve public understanding, leading to more proactive health behaviors. Addressing negative attitudes and fears associated with monkeypox is equally crucial. Misconceptions and stigma can hinder individuals from seeking medical help or following public health guidelines. By fostering a positive and informed attitude toward monkeypox, healthcare providers and policymakers can reduce the barriers to effective disease management. This involves not only providing factual information but also engaging with communities to understand their concerns and addressing them through culturally sensitive communication and support.
